# Sexually Dimorphic Effect of Genistein on Hypothalamic Neuronal Differentiation in Vitro

**DOI:** 10.3390/ijms20102465

**Published:** 2019-05-18

**Authors:** Marilena Marraudino, Alice Farinetti, Maria-Angeles Arevalo, Stefano Gotti, GianCarlo Panzica, Luis-Miguel Garcia-Segura

**Affiliations:** 1 Neuroscience Institute Cavalieri-Ottolenghi (NICO), 10043 Orbassano, Italy; alice.farinetti@unito.it (A.F.); stefano.gotti@unito.it (S.G.); giancarlo.panzica@unito.it (G.P.); 2 Department of Neuroscience, University of Torino, 10126 Torino, Italy; 3 Instituto Cajal, CSIC, Avenida Doctor Arce 37, E-28002 Madrid, Spain; arevalo@cajal.csic.es (M.-A.A.); lmgs@cajal.csic.es (L.-M.G.-S.); 4 Centro de Investigación Biomédica en Red de Fragilidad y Envejecimiento Saludable (CIBERFES), Instituto de Salud Carlos III, E-28029 Madrid, Spain

**Keywords:** genistein, sex difference, hypothalamic neurons, neuritogenesis, estrogen receptors

## Abstract

Developmental actions of estradiol in the hypothalamus are well characterized. This hormone generates sex differences in the development of hypothalamic neuronal circuits controlling neuroendocrine events, feeding, growth, reproduction and behavior. In vitro, estradiol promotes sexually dimorphic effects on hypothalamic neuritogenesis. Previous studies have shown that developmental actions of the phytoestrogen genistein result in permanent sexually dimorphic effects in some behaviors and neural circuits in vivo. In the present study, we have explored if genistein, like estradiol, affects neuritogenesis in primary hypothalamic neurons and investigated the estrogen receptors implicated in this action. Hypothalamic neuronal cultures, obtained from male or female embryonic day 14 (E14) CD1 mice, were treated with genistein (0.1 µM, 0.5 µM or 1 µM) or vehicle. Under basal conditions, female neurons had longer primary neurites, higher number of secondary neurites and higher neuritic arborization compared to male neurons. The treatment with genistein increased neuritic arborization and the number of primary neurites and decreased the number of secondary neurites in female neurons, but not in male neurons. In contrast, genistein resulted in a significant increase in primary neuritic length in male neurons, but not in female neurons. The use of selective estrogen receptor antagonists suggests that estrogen receptor α, estrogen receptor β and G-protein-coupled estrogen receptors are involved in the neuritogenic action of genistein. In summary, these findings indicate that genistein exerts sexually dimorphic actions on the development of hypothalamic neurons, altering the normal pattern of sex differences in neuritogenesis.

## 1. Introduction

Some natural nonsteroidal molecules, contained in vegetal species that are components of the diet of humans and animals (i.e., leguminous, including soy), show estrogenic activity and are therefore called phytoestrogens [1]. These compounds have a weak to moderate affinity for estrogen receptors (ERs) and are therefore considered xenoestrogens and included in the list of endocrine disrupting chemicals [2]. The more common phytoestrogens in the human diet are the flavonoids, including cumestrol, daidzein and genistein, which is probably the most abundant one [3]. 

Phytoestrogens, in particular genistein, which is able to interact with the estrogen-dependent neural pathways in complex and multidirectional ways [4,5], may interfere with the endocrine system, leading to permanent alterations of estrogen sensitive circuits [6]. Due to its ability of binding ERs, genistein is generally considered as a beneficial molecule, being used as a natural substitute for endogenous hormones in some physiological situations (i.e., menopause). However, recent studies in vivo demonstrated that genistein treatment during development exerts a permanent and sexually dimorphic alteration of some behaviors and neural circuits [7,8]. Previous studies have also shown that estradiol exerts sexually dimorphic actions on neurite outgrowth and elongation in developing hypothalamic neurons [9,10]. In this study, we have explored if genistein, like estradiol, exerts sexually dimorphic actions on neuritogenesis in hypothalamic neurons and whether ERs are involved in these actions. 

## 2. Results

### 2.1. Effects of Genistein on the Number and the Length of Primary Neurites

**Figure 1:**
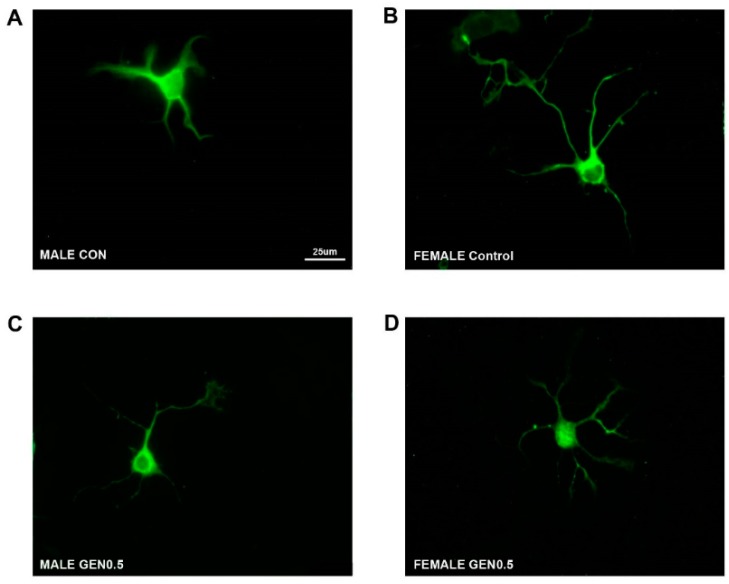
Representative examples of microtubule associated protein-2 (MAP2) immunoreactive primary hypothalamic neurons. (**A**) Male control neuron; (**B**) female control neuron; (**C**) male neuron treated with 0.5 µM genistein; (**D**) female neuron treated with 0.5 µM genistein. Scale bar = 25 μm.

**Figure 2:**
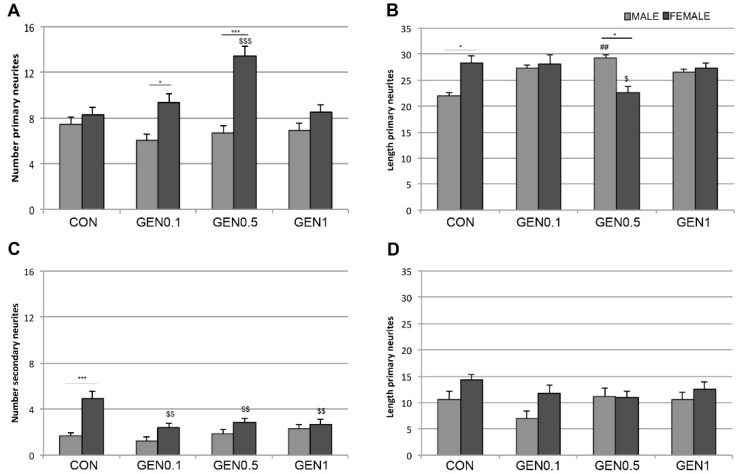
Number and length of primary and secondary neurites. (**A**) Number of primary neurites in male and female cultures treated with control medium (CON) or genistein 0.1 µM (GEN0.1), 0.5 µM (GEN0.5) and 1 µM (GEN1). (**B**) Length of primary neurites in male and female cultures treated with control medium (CON) or genistein 0.1 µM (GEN0.1), 0.5 µM (GEN0.5) and 1 µM (GEN1). (**C**) Number of secondary neurites in male and female cultures treated with control medium (CON) or genistein 0.1 µM (GEN0.1), 0.5 µM (GEN0.5) and 1 µM (GEN1). (**D**) Length of secondary neurites in male and female cultures treated with control medium (CON) or genistein 0.1 µM (GEN0.1), 0.5 µM (GEN0.5) and 1 µM (GEN1). Data are the mean ± SEM of 30 hypothalamic neurons. Significant differences with the post hoc Tukey test: Male vs female * *p* < 0.05, *** *p* < 0.001; female control vs female treated ^$^ *p* < 0.05; ^$$^ *p* < 0.01; ^$$$^ *p* < 0.001; male control vs male treated ^##^ *p* < 0.01.

Figure 1 shows representative examples of cultured hypothalamic neurons immunostained for microtubule associated protein-2 (MAP2). The number of primary neurites in male and female cultures treated with control medium or genistein (0.1, 0.5 and 1 µM) is represented in Figure 2A. No significant differences in the number of primary neurites were detected in the cultures incubated with control medium. In contrast, the treatment with genistein resulted in a sexually dimorphic effect on the number of primary neurites. As shown in Figure 2A, genistein increased the number of primary neurites in female neurons in a dose-dependent way, reaching a maximal effect at 0.5 µM and losing its effect at 1 µM. Two-way analysis of variance (ANOVA) revealed an effect of sex (*F* = 41.694; *p* = 0.001) and treatment (*F* = 5.664; *p* = 0.001) and an interaction between sex and treatment (*F* = 7.433; *p* = 0.001) in the number of primary neurites. The comparisons by post hoc Tukey test revealed a significant effect of the treatments with 0.1 µM (*p* = 0.02) and 0.5 µM (*p* = 0.001) genistein in the number of primary neurites in female hypothalamic cultures. In contrast, genistein treatment did not affect the number of primary neurites in male neurons (Figure 2A).

The length of primary neurites in male and female cultures treated with control medium or genistein (0.1, 0.5 and 1 µM) is represented in Figure 2B. This parameter was significantly higher in female neurons compared to male neurons under control conditions. Treatment with genistein abolished this sex difference, increasing the length of primary neurites in male cultures. Two-way ANOVA showed a significant effect of the interaction of sex and treatment (*F* = 6.220; *p* = 0.001), but not a significant effect of sex (*F* = 0.062; *p* = 0.804) or treatment (*F* = 1.080; *p* = 0.358). Post-hoc analysis revealed a significant sex difference in primary neuritic length (*p* = 0.05) in control cultures, showing female neurons longer neurites than male neurons. Treatment with 0.5 µM genistein resulted in a significant increase in primary neuritic length in male neurons (*p* = 0.018) compared to control male neurons. Under these conditions, male neurons showed longer primary neurites than female neurons (*p* = 0.041). In contrast, genistein significantly decreased the length of primary neurites in female neurons (*p* = 0.031) (Figure 2B).

### 2.2. Effects of Genistein on the Number and the Length of Secondary Neurites

The number of secondary neurites in male and female cultures treated with control medium or genistein (0.1, 0.5 and 1 µM) is represented in Figure 2C. Female neurons showed a higher number of secondary neurites than male neurons under control conditions. This sex difference was abolished by genistein treatment, which decreased the number of secondary neurites in female neurons. Two-way ANOVA revealed a significant effect of the treatment (*F* = 4.272; *p* = 0.006). As shown by the post hoc analysis, the number of secondary neurites was higher in female control neurons in comparison with male control neurons (*p* = 0.001). The three genistein concentrations studied resulted in a significant decrease in the number of secondary neurites in female neurons (0.1 µM, *p* = 0.001; 0.5 µM, *p* = 0.01 and 1 µM, *p* = 0.003) (Figure 2C). In contrast, genistein treatment did not significantly affect the number of secondary neurites in male neurons.

The length of secondary neurites in male and female cultures treated with control medium or genistein (0.1, 0.5 and 1 µM) is represented in Figure 2D. No significant differences were detected in the length of secondary neurites between male and female neurons and this parameter was not significantly modified by genistein.

### 2.3. Effects of Genistein on Neuritic Arborizations

**Figure 3:**
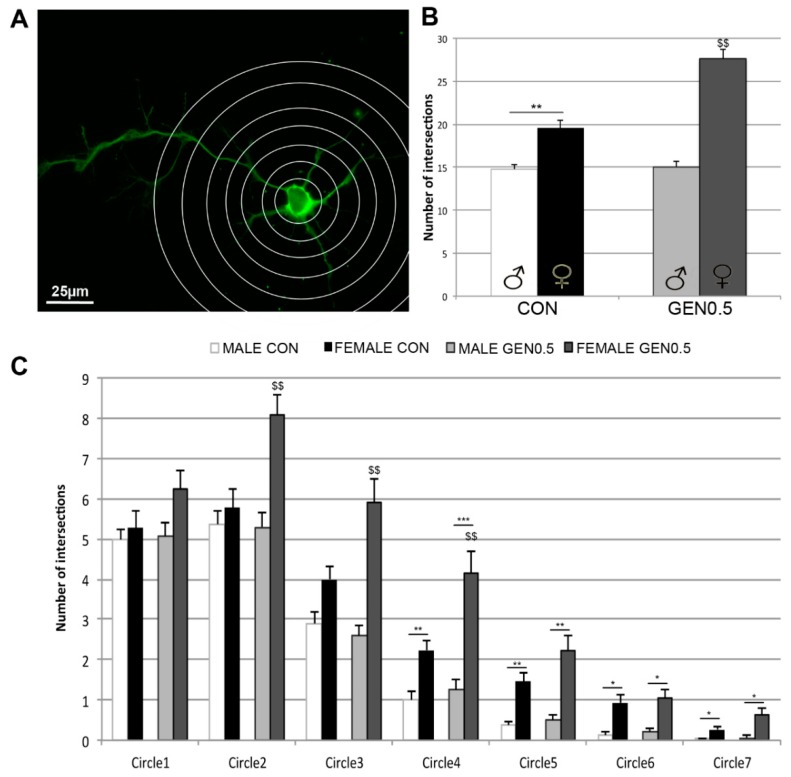
Neuritic arborization assessed with Sholl analysis. (**A**) Representative example of a neuron with the grid used for the Sholl analysis. The image corresponds to a female neuron treated with 0.5 µM genistein. Scale bar = 25 μm. (**B**) Number of intersections of the neurites with the grid in male and female control (CON) neurons and in male and female neurons treated with 0.5 µM genistein (GEN0.5). (**C**) Analysis of intersections in function of the distance to the cell soma in control male (MALE CON) and female (FEMALE CON) neurons and in male (MALE GEN0.5) and female (FEMALE GEN0.5) neurons treated with 0.5 µM genistein. Data are the mean ± SEM of 30 hypothalamic neurons. Significant differences with the post hoc Tukey test: Male vs female * *p* < 0.05, ** *p* < 0.01, *** *p* < 0.001; female control vs female treated ^$$^ *p* < 0.01.

Sholl analysis of neuritic arborization was performed in control cultures and in cultures treated with 0.5 µM genistein, following the previous results indicating a maximal effect of genistein at this concentration on the number and length of primary and secondary neurites. An example of the grid used for the analysis is shown in Figure 3A. The total number of intersections of the lines of the grid with the neurites in male and female neurons under control conditions and after treatment with 0.5 µM genistein is shown in Figure 3B. One-way ANOVA analysis revealed a significant effect of the treatment (*F* = 4.001; *p* = 0.048). Under control conditions, female neurons showed an increased neuritic arborization compared to male neurons (*p* = 0.007). This sex difference was amplified after genistein treatment, which significantly increased neuritic arborization in female neurons (*p* = 0.001), but did not affect this parameter in male neurons (Figure 3B). Analysis of intersections in function of the distance to the cell soma is represented in Figure 3C. This analysis revealed basal sex differences in circles 4–7, while the effect of genistein on female neurons was detected in circles 2–4 (Figure 3C).

### 2.4. Effects of Estrogen Receptor Antagonists on Neuritic Arborizations

**Figure 4:**
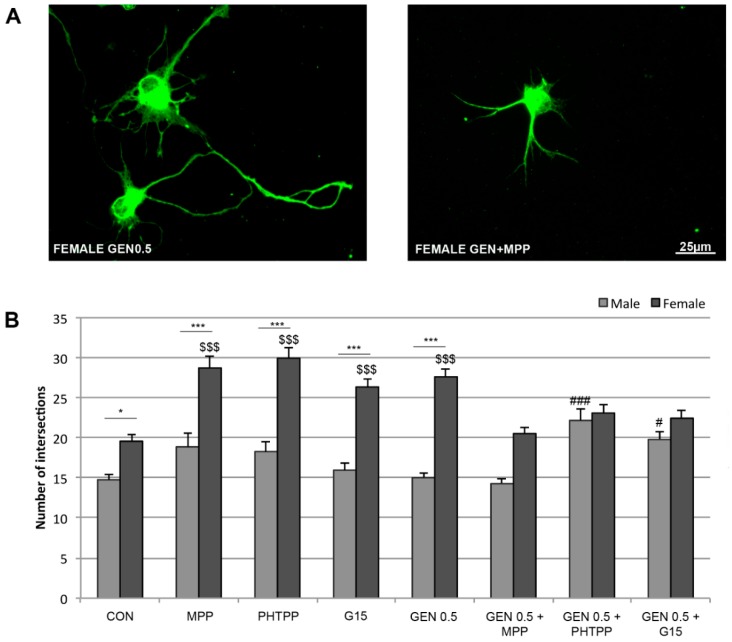
Effects of estrogen receptor antagonists on neuritic arborization. (**A**) Representative examples of female neurons treated with 0.5 µM genistein (left panel) or 0.5 µM genistein and the ERα antagonist MPP. (**B**) Effects of antagonists for ERα (MPP), ERβ (PHTPP) or GPER (G15) on the number of neurite intersections assessed by Sholl grid. CON, control neurons treated with vehicle; MPP, control neurons treated with MPP; PHTPP, control neurons treated with PHTPP; G15, control neurons treated with G15; GEN 0.5, neurons treated with 0.5 µM genistein; GEN 0.5 + MPP, neurons treated with 0.5 µM genistein and MPP; GEN 0.5 + PHTPP, neurons treated with 0.5 µM genistein and PHTPP; GEN 0.5 + G15, neurons treated with 0.5 µM genistein and G15. Data are the mean ± SEM of 30 hypothalamic neurons. Significant differences with the post hoc Tukey test: male vs female * *p* < 0.05, *** *p* < 0.001; female control vs female treated ^$$$^ *p* < 0.001; male control vs male treated ^#^ *p* < 0.05, ^###^ *p* < 0.001.

To determine the possible role of ERs in the neuritogenic action of genistein, the effects of antagonists for ERα (MPP), ERβ (PHTPP) or GPER (G15) were assessed on neuronal morphology in neurons treated with control medium or 0.5 μM genistein. Figure 4 shows the results of the Sholl analysis. One-way ANOVA analysis revealed a significant effect of the treatment (*F* = 22.152; *p* = 0.001). In agreement with the previous experiment, genistein increased the number of intersections in the Sholl analysis in female neurons (*p* = 0.001). This effect was blocked by the ERα antagonist MPP, by the ERβ antagonist PHTPP and by the GPER antagonist G15, suggesting that the effect of genistein on neuritic arborization in female neurons is mediated by ERs. However, some additional interesting effects of the ER antagonists were observed. Both MPP, PHTPP and G15 significantly increased neuritic arborization in control female neurons, but did not affect neuritic arborizations in control male neurons. In addition, genistein increased neuritic arborization in male neurons in the presence of PHTPP or G15.

## 3. Discussion

In this study, we analyzed, for the first time, the effect of genistein on hypothalamic neurons in culture. Our present results confirm previous findings on the existence of sex differences in neuritogenesis of primary hypothalamic neurons obtained from E14 mice embryos [10]. As reported by previous studies, control female neurons showed a higher neuritic arborization than control male neurons, assessed by Sholl analysis. We also showed a sex difference in the response to genistein treatment. In fact, only in female neurons, genistein induced a significant increase in the arborization of neuronal processes, enlarging the natural sex difference in neuritogenesis.

Our present findings in vitro, showing sex differences in the effect of genistein on the neuritic arbor of developing hypothalamic neurons, extend previous studies in vivo, showing that developmental actions of genistein exert alterations of some behaviors and neuronal circuits in a permanent and sexually different manner. In fact, prenatal or postnatal administration of genistein in rodents interferes with anxiety-related behaviors [8] and with neuronal nitric oxide synthase (nNOS), vasopressin (AVP) and kisspeptin (Kiss) pathways in the adult [7]. Our present results suggest that these developmental actions of genistein may include alterations in neuritogenesis, which may impact on the maturation of specific neuronal circuits with long-term and sexually dimorphic functional consequences.

Early studies by Kuiper et al. demonstrated that genistein acts as an agonist on ERα and ERβ, but has a higher affinity for ERβ, based on solubilized receptor-binding assay [4]. In addition, the ability of genistein to mimic the action of estradiol on NMDA-stimulated AVP and oxytocin release from hypothalamus suggests that its effect on these parameters is mediated by ERβ [5]. However, our present results, using selective ER antagonists, suggest that the effect of genistein on neuritic arborization in female neurons is mediated by both ERα, ERβ and GPER. Therefore, the role of the different ER subtypes on the effects of genistein may depend on the cell system or the parameter considered and may be different on developing and adult neurons. In addition, the role of ERs on the actions of genistein may also be different in male and female neurons. Indeed, our findings indicate that, in contrast to what was observed for female neurons, in the presence of the antagonist for ERβ or the antagonist for GPER, but not in the presence of the antagonist for ERα, genistein induced an increase in neuritic branching of male neurons. This finding may suggest that genistein is exerting an inhibition on neuritic arborization in male neurons through ERβ or GPER. Since, genistein alone did not affect neuritic arborization in male neurons, this hypothetical inhibitory action on neuritic arborization via ERβ and GPER should be compensated by a stimulatory action through other mechanisms. However, genistein did not decrease neuritic arborization in male neurons in the presence of the ERα antagonist, suggesting that the activation of ERα is not compensating for the effects on ERβ and GPER. Further studies, should therefore determine the mechanisms involved in the facilitation of the neuritogenic effect of genistein on male neurons when ERβ or GPER are antagonized.

The analysis of the effect of selective ER antagonists also suggests a role of ERs in the generation of basal sex differences in neuritic arborization. Thus, neuritic arborization was increased in control female neurons by ERα, ERβ or GPER antagonist. A possible explanation is that endogenous estradiol produced by female neurons [11] exerts an inhibitory action on neuritic arborization by acting separately on each ER subtype. However, previous studies indicate that the global effect of endogenous estradiol in female neurons is to promote, and not to inhibit, neuritogenesis [11,12]. Therefore, further experiments are necessary to clarify the effect of ER antagonists on neuritogenesis and to explore the nature of the interactions between the different ERs in the control of neuronal morphology. A good candidate could be Neurogenin 3 (Ngn3), a Notch-regulated gene, strongly involved in the generation of sexual differences in hypothalamic and hippocampal neuritogenesis (as well described in review [12]). In fact, Ngn3 expression is dimorphic, higher in primary female hypothalamic [10] and hippocampal [11] cultures than male. Moreover, treatments with estradiol increased levels of Ngn3 only in male neurons, promoting neuritogenesis and abolishing dimorphism in neuronal development and Ngn3 expression [10]. Therefore, it could be very interesting to study the effect of genistein treatment on Ngn3 expression, as possible mechanism that could be involved in determining the sex differences in neuritogenesis of primary hypothalamic neurons observed in this study.

The values of neuritic arborization obtained with the Sholl analysis reflect the combination of the number, length and branching of neurites. The morphometric analysis of the number and length of primary and secondary neurites in the hypothalamic cultures suggest that genistein is regulating in a sexually dimorphic way the mechanisms that control neurite formation, neuritic length and neuritic branching. Thus, genistein increased the number of primary neurites, but reduced its length and branching (i.e., number of secondary neurites) in female neurons. In contrast, genistein increased primary neuritic length in male neurons, without affecting its number and branching. In addition, our findings suggest that basal sex differences in neuritogenesis depend on an increased length and branching of primary neurites in female neurons. These different basal sex differences and effects of genistein on primary neuritic number, length and branching are in agreement with the observation that genistein increased the intersections of female neurites with the internal circles (2–4) of the Sholl grid in female neurons, which probably reflect the increase in the number of primary neurites. In contrast, basal sex differences in neuritic arborization were detected in the outer circles (4–7), probably reflecting differences in primary neuritic length and branching.

In summary, our findings indicate that genistein exerts sexually dimorphic actions on the development of hypothalamic neurons, altering some specific parameters of the neuritogenic process in female neurons and different parameters in male neurons. These developmental actions of genistein interfere with the normal sex differentiation of hypothalamic neurons and may therefore have relevant functional consequences. Further studies are now necessary to determine the molecular mechanisms involved in the sexually dimorphic effects of genistein on neuritogenesis and to characterize these effects in vivo.

## 4. Materials and Methods

### 4.1. Animals

The embryos used for this study were obtained from CD1 mice raised at the Cajal Institute (Madrid, Spain). The day of vaginal plug was defined as E0. All procedures for handling and killing the animals used in this study were in accordance with the European Commission guidelines (86/609/CEE and 2010/63/UE) and the Spanish Government Directive (R.D. 1201/2005). The Cajal Institute Ethic Committee of Animal Experimentation approved the experimental procedures (CEEA-IC 2015/039/CEI 3/20150622; 22-06-2015).

### 4.2. Hypothalamic Neuronal Cultures and Cell Treatments

Hypothalamic neurons were obtained from male and female mouse embryos at embryonic day 14 (E14) and were cultured separately according to the sex of fetal donors. Male fetuses were identified under a dissecting microscope by the presence of the spermatic artery on the developing gonad [10]. The brain was dissected out and the meninges were removed. Then, the ventromedial hypothalamic region, delimited by the optic chiasm, the lateral hypothalamic sulcus and the mammillary bodies, was dissected out from the diencephalon. The soft block of tissue was dissociated to single cells after digestion for 15 min at 37 °C with 0.5% trypsin (Worthington Biochemicals, Freehold, NJ, USA) and DNaseI (Sigma-Aldrich Co. St. Louis, MO, USA) and washed in Ca2+/Mg2+-free Hank’s Buffered Salt Solution. Neurons were counted and plated on glass coverslips coated with poly-L-lysine (Sigma-Aldrich) at a density of 200 cells/mm^2^. Cells were then cultured for 3 days in phenol-red-free Neurobasal supplemented with B-27 and GlutaMAXI (Invitrogen, Crewe, UK).

For the treatments, in a first experiment, male and female cultures were incubated for 1 day with genistein (0.1 µM, 0.5 µM or 1 µM; Sigma-Aldrich) or vehicle. After the first analysis of the effects of these three different doses of genistein, we treated additional female and male hypothalamic cultures for 1 day with selective estrogen receptor antagonist: The selective ERα antagonist 1,3-bis (4-hydroxyphenyl)-4-methyl-5-[4-(2-piperidinyl-ethoxy) phenol]-1H–pyrazole dihydrochloride (MPP; 10^−8^ M); the selective ERβ antagonist 4-[2-phenyl-5,7-bis (trifluoromethyl) pyrazolo [1,5-a]pyrimidin-3-yl]phenol (PHTPP;10^−8^ M) and the selective GPER antagonist G15 (10^−8^ M) dissolved in Neurobasal medium in combination with or without 0.5 µM genistein.

### 4.3. Immunocytochemistry

Cells were fixed for 20 min at room temperature in 4% paraformaldehyde and permeabilized for 4 min with 0.12% Triton-X plus 0.12% gelatin in phosphate-buffered saline (PBS). Cells were then washed with PBS/gelatin and incubated for 1 h with a chicken polyclonal antibody for microtubule associated protein-2 (MAP2; diluted 1:100 in PBS/gelatin; AB15452, Sigma-Aldrich). After washing in PBS, cells were incubated for 45 min with anti-chicken Alexa Fluor^®^ 488 (492/520 nm; AB2340375) secondary antibody made in donkey (diluted 1:500 in PBS/gelatin). Cell nuclei were stained with 4′,6-diamidino-2-phenylindole (DAPI).

### 4.4. Analysis of Neuronal Processes

Neurites were traced using NeuronJ plugin for ImageJ (freely available at https://imagej.nih.gov/ij/). A specific color was assigned for primary neurites (those emerging directly from the soma) and for secondary neuritic processes (branches emerging from primary neurites) [13]. Neuritic number and length was measured with the ImageJ program and a text file containing measurement data of neurite number and length was generated for each labeled neuron. Moreover, neuritic arborization was assessed with the method of Sholl [14], using a grid of 7 concentric circles with increasing radius of 20 μm, placing the innermost circle over the soma. The morphometric analysis of neuronal processes and the Sholl analysis were done using the images of MAP2 immunoreactive neurons at a magnification of 400× and in 30 cells randomly taken from three different cultures per each experimental condition.

### 4.5. Statistical Analysis

The statistical analysis was performed using the SPSS 25.0 statistic software (SPSS Inc, Chicago, IL, USA), and was undertaken using two- or one-way ANOVA and with post hoc Tukey test; values of *p* ≤ 0.05 were considered significant. Data are represented as mean ± SEM values.
